# Global Warming and the Elderly: A Socio-Ecological Framework

**DOI:** 10.3390/ijerph23020164

**Published:** 2026-01-28

**Authors:** Nina Hanenson Russin, Matthew P. Martin, Megan McElhinny

**Affiliations:** 1 College of Health Solutions, Arizona State University, Phoenix, AZ 85004, USA; 2 Valleywise Health Medical Center, Phoenix, AZ 85008, USA; 3 Department of Emergency Medicine, Creighton University, Phoenix Campus, Phoenix, AZ 85012, USA; 4 Department of Emergency Medicine, University of Arizona, Phoenix Campus, Phoenix, AZ 85004, USA

**Keywords:** climate change, global warming, older adults, elderly, heat-related illness

## Abstract

**Highlights:**

**Public health relevance—How does this work relate to a public health issue?**

- Two global trends, including the aging of populations worldwide and the acceleration of climate change with increasingly frequent extreme weather events, may contribute to a healthcare crisis toward the end of the current century.
- Heat-related mortality among adults ages 60 and above has increased by 167% since the 1990s, with global warming now occurring at three times the rate of the pre-industrial era.

**Public health significance—Why is this work of significance to public health?**

- The study proposes a socio-ecological framework that considers both drivers and mitigation strategies for addressing heat-related illness in the elderly from the perspectives of global climate and policy, social determinants of health, and the individual.
- It will be essential to improve heat emergency warning systems and critical care management for older adults to prevent unnecessary morbidity and mortality in the wake of global warming.

**Public health implications—What are the key implications or messages for practitioners, policymakers and/or researchers in public health?**

- Healthcare providers play an essential role in reducing the risks for heat-related illness through patient education regarding the symptoms of heat exhaustion and heat stroke.
- Advocacy for changes in public policy related to the built environment will be key to this effort, including addressing the problems of urban heat islands, providing public cooling centers during heat emergencies, and promoting the use of universal design concepts to improve pedestrian pathways for persons with limited mobility

**Abstract:**

**Problem Statement:** Two global trends, including aging populations and the acceleration of global warming, are increasing the risk of heat-related illness, challenging the health of populations, and the sustainability of healthcare systems. Global warming refers to the increase in the Earth’s average surface temperature, generally attributed to the greenhouse effect, which is occurring at three times the rate of the pre-industrial era. The global population of older adults, defined here as individuals aged 60 and over, is expected to reach over 2 billion by mid-century. This population is particularly vulnerable to heat-related illness, specifically disruption of thermoregulation from excessive exposure to environmental heat due to metabolic and cognitive changes associated with aging. **Objectives:** This review examines heat-related illness and its impact on older adults within a socio-ecological framework, considering both drivers and mitigation strategies related to global warming, the built environment, social determinants of health, healthcare system responses, and the individual. The authors were motivated to create a conceptual model within this framework drawing on their lived experiences as healthcare providers interacting with older adults in a large urban area of the southwestern US, known for its extreme heat and extensive heat island effects. Based on this framework, the authors suggest actionable strategies supported by the literature to reduce the risks of morbidity and mortality. **Methods:** The literature search utilized a wide lens to identify evidence supporting various aspects of the hypothesized framework. In this sense, this review differs from systematic and scoping reviews, which seek a complete synthesis of the available literature or a mapping of the evidence. The first author conducted the literature search and synthesis, while the second and third authors reviewed and added publications to the initial search and conceptualized the socio-ecological framework. **Discussion:** This study is unique in its focus on a global trend that threatens the well-being of a growing population. The population health focus underscores social determinants of health and limitations of existing healthcare systems to guide healthcare providers in reducing older adults’ vulnerability to heat-related illness. This includes patient education regarding age-related declines in extreme heat tolerance, safe and unsafe physical activity habits, the impact of prescription drugs on heat tolerance, and, importantly, identifying the symptoms of heatstroke, which is a medical emergency. Additional strategies for improving survivability and quality of life for this vulnerable population include improved emergency response systems, better social support, and closer attention to evidence-based treatment for heat-related health conditions.

## 1. Introduction

Global warming in the 21st century is occurring at three times the rate of the pre-industrial era, with the ten warmest years in historical records occurring over the past decade [1]. At the same time, the number of older adults will increase to about 2.1 billion individuals by the year 2050, representing 22% of the world’s population [2]. Older adults are more susceptible to heat-related illness [3], defined as a disruption of thermoregulation due to excessive heat [4], caused by age-related cardiometabolic and cognitive changes [5,6,7]. The intersection of these two trends creates the potential for a “perfect storm,” a healthcare crisis with significant impact on both the health and wellness of populations and healthcare infrastructure.

Heat-related mortality among individuals aged 65 and over has increased by 167% compared to the 1990s, which has been attributed to climate change [8]. Chronic diseases that may impact heat tolerance include cardiovascular disease, kidney disease, diabetes, and dementia [9]. A systematic review and meta-analysis involving over 729,000 older adults found that 46% of older adults worldwide live with at least two chronic conditions [10]. Resilience to heat may also be impacted by medications used to manage chronic disease, such as beta blockers, antipsychotics, and anticholinergics [3,9]. Social determinants of health, including social isolation [11], food insecurity, suboptimal housing, and limited transportation options, intersect with limited awareness about the signs and symptoms of heat illness, which exacerbates the problem [3].

This narrative was motivated by the authors’ lived experience in a major urban area in the Southwestern US, which has been cited for its extremely high summer temperatures (frequently averaging 103–106 °F (39–41 °C) [12]. In addition, the area has an unusually high percentage of older adult residents due to its mild winters. Therefore, the heat-related conditions described in this review, including heat stroke and pavement contact burns, are relatively common. The focus is on population health approaches to decreasing the impact of extreme temperatures on this group of individuals. A socio-ecological framework contextualizes the relationship between the drivers of heat-related illness and strategies for lessening its impact on older adults.

## 2. Methods

This review utilizes a broad lens to give an overview of the two trends described above. Therefore, the search strategy differed from a systematic review, which would focus on a systematic search of evidence on a narrower topic, or a scoping review (also narrower in focus) that would seek a complete mapping of the evidence. The first author searched peer-reviewed literature using the terms ((“climate change” OR “global warming”) AND (“older adults” OR elderly OR “aging population”)) AND (“heat related illness”) across two databases (Google Scholar and PubMed). Gray literature (government and agency publications, expert commentary) was utilized to add information on insurance reimbursement for medications, universal design and housing insecurity, heat wave statistics, and the physiology of heat-related illness. Following review, additional searches were conducted utilizing the same databases to extract studies focusing on the impact of race, ethnicity, and socioeconomic status on heat illness vulnerability, and to clarify definitions of climatological terminology utilized in the narrative. Both the second and third authors reviewed the search strategy and references.

### Research Question

Given known increases in global surface temperatures and the aging of the global population, what population health approaches can protect the health and quality of life of persons ages 60+ who are at increased risk of heat-related illnesses?

Inclusion criteria included peer-reviewed English language-only literature with no lower date limit specified, as well as the expert commentary mentioned above. Other types of gray literature, such as conference abstracts, were not included. The second and third authors reviewed the search, added literature not identified by the first author, and developed the socio-ecological framework for drivers of heat-related illness (HRI) and strategies for managing HRI in older adults.

## 3. Results

Section 3 begins with a descriptive summary of the included literature, with Table 1, Table 2 and Table 3 outlining study origins, types, and topical focus. The section then synthesizes key themes that emerged across studies, including physiological vulnerability, environmental and structural determinants of heat risk, and population health planning considerations for older adults. Table 1 lists the sources extracted for this study by the authors’ countries of origin and the primary focus, relative to this discussion. Of the 75 total studies cited below, 40 originate from inside the USA, while 35 originate from other countries (Australia, Belgium, Canada, China, Denmark, Georgia, India, Italy, Japan, Malaysia, New Zealand, Pakistan, Saudi Arabia, Singapore, South Africa, Spain, Switzerland, and the UK).

### 3.1. A Population Health Approach at the Intersection of Two Global Trends

Data extracted for this narrative review fall into two broad categories: research conducted on the physiology of healthy aging, chronic diseases common among older adult populations and health-related factors contributing to their increased vulnerability to HRI [2,3,4,5,6,8,9,10,16,17,18,21,23,30,32,37,38,39,40,44,46,47,49,50,53,54,62,64,65,66,69,75]. Table 2 organizes the “physiology” publications (n = 32) by source or study type and narrative focus. This list is predominantly composed of synthesis and conceptual sources (e.g., narrative reviews, systematic reviews, perspectives, and editorials), supplemented by a smaller number of empirical studies, including observational analyses and clinical trials. Government reports and organizational guidance documents also featured prominently.

Environmental factors, including structural, social, economic, and policy issues, as well as heat action plans and healthcare systems [7,11,12,13,14,15,19,20,22,24,25,26,27,28,29,31,34,36,41,42,43,48,52,55,56,57,58,59,60,61,63,67,70,73,74,76].

Table 3 summarizes research (n = 38) on environmental factors by study type and narrative focus. This list was also dominated by synthesis studies, including narrative and systematic reviews. Empirical studies and trials were comparatively limited, while government and policy-oriented sources featured prominently.

### 3.2. Heat Health Terminology

While extracted studies examined the physiology of heat-related illness, considering both healthy aging and chronic disease, there was disagreement over the use of specific terminology, leading to gaps in the research. For example, some studies considered older adults to be those over age 52 [40] while others used a baseline of 60+ [53], 65+ [8,35,49], 70 [6], 71 [65], and 75+ [21]. One resource [50] divided “older adult” populations into age groups, beginning at age 45. This heterogeneity makes it difficult to compare findings across studies. Terminology surrounding climate change and global warming was also inconsistent. In some cases, the term “climate change” was used to refer to both global warming (increases in the earth’s surface temperatures) and climate change (extreme weather events resulting from global warming) [8]. In other cases, the term “climate change” refers to global warming [1].

### 3.3. Physiology and Climatology

Ideally, the effects of heat and humidity on human physiology should be described specifically, since arid heat affects the body differently than humid heat, because sweating cools the body more efficiently in the former than in the latter [54]. Yet most of the extracted studies failed to mention the specific temperature and humidity levels associated with heat-related illness. For example, in their discussion of emergency department visits in the US, Wu and colleagues [9] describe heat stroke using standard physiologic descriptors (core temperature, dry skin, delirium and coma) but are much less specific about predisposing factors (high ambient temperature, intense solar radiation), with the most likely reason being limitations within medical records. In their systematic review of heat tolerance in older adults, Núñez-Rodriguez et al. [53] state that both high ambient temperature and high humidity contribute to risk of heat-related illnesses, but they fail to explain the relationship between the two. Similarly, studies on aging and thermoregulatory control in older adults [16,49] focus on specific physiological changes (regulation of body temperature, sweat rate, skin blood flow, cardiovascular response, etc.). However, discussions regarding environmental conditions that predispose persons to HRI, such as the interaction of heat and humidity, are much less specific. Pragmatic studies that examine both climactic conditions and physiologic changes in detail could be extremely valuable for improving heat action plans and emergency response protocols.

### 3.4. Population Health Planning

In 2015, the World Meteorological Association and the World Health Organization [77] published guidance on the development of heat action plans and emergency warning systems, based primarily on information from the USA and Europe, based largely on research during the first decade of the current century. We discuss some of these action plans in this study. While heat action plans have become commonplace in many first-world nations, the aging of populations in low- and middle-income countries (see below) underscores the importance of such planning in these emerging population centers (Africa, India, China). A recent study on urban heat action plans in India [63] states that while such planning has made significant strides, it is still in its infancy, with a focus on relief measures rather than proactive planning. This is an important area for future research and development.

### 3.5. Low- and Middle-Income Nations

According to the World Health Organization [2], the highest percentage of older adults currently reside in first-world nations, with Japan, where 30% of the population is over age 60, leading the way. However, by 2050, the WHO predicts that 2/3 of the global older adult population will live in low-and middle-income nations [2]. However, the bulk of current research on this topic is taking place in North America, as reflected in a 2025 systematic review on heat tolerance in older adults [53]. For the latter study, 41% of cited research came from North America, followed by Europe, China, and Oceania [53]. A second systematic review [61] also notes the paucity of research in low- and middle-income countries (LMICs), with most work conducted in first-world nations.

Our literature review yielded similar findings, with the bulk of studies originating in first-world nations in North America, Europe, Australia, and New Zealand. While our research included multiple studies from Malaysia and China, it will be important for further research to take place in low- and middle-income nations as their demographics shift. Only one of the extracted studies came from Africa, two were conducted in Pakistan, and one was conducted in Saudi Arabia. Specific areas need addressing with regard to developing nations: emergency preparedness plans, the potential impact of community health workers to reduce the risk of heat-related morbidity and mortality, specifically among older adults in these areas, existing educational programs and the potential for enhanced education, and importantly, the need for more rigorous recording of heat waves, and their impact on older adult residents. Based on our research, this trend appears to be changing, with more studies being conducted in low-and-middle-income nations.

## 4. Discussion

This review examines the effects of extreme heat associated with global warming on heat-related illness among older adults, integrating physiological and environmental perspectives. The discussion focuses specifically on heat-related conditions commonly observed in this population and does not address other climate-related health threats. We synthesized evidence on age-related physiological vulnerability, the role of chronic disease and medication use in heat tolerance, and the ways in which social, economic, and environmental conditions shape risk. The discussion then expands to population health planning, including the effectiveness of heat action plans, emergency warning systems, and system-level interventions, with particular attention to gaps in preparedness for older adults in low- and middle-income countries.

### 4.1. Population Aging and Physiological Vulnerability

#### 4.1.1. Aging of Global Populations

Globally, populations are aging, with the number of older adults expected to increase significantly by the middle of the 21st century. China is considered to have the fastest-growing population of older adults among developing countries [76], having increased from 13.32% of the population in 2010 to 18.73% (264 million individuals) in 2020 [36]. The US Census Bureau estimates that by 2050, the global population of individuals ages 65 and older will reach 1.6 billion people [76]. The United Nations Population Division has projected that the population over age 60 will increase to over 2 billion by mid-century, representing 22% of the global population [30]. These demographic changes in the face of increasingly rapid climate change present a unique challenge.

#### 4.1.2. Physical and Cognitive Decline in Healthy Aging and Chronic Disease

Symptoms reflecting decline in physical and cognitive function can be divided into those associated with healthy aging and a second group related to chronic disease. Physiological changes that occur with healthy aging include declines in bone density and muscle mass, changes in vision and hearing acuity, and, in some cases, cognitive slowing. While resting heart rate may remain unchanged, maximum heart rate, maximum cardiac output, and maximum and relative maximum oxygen uptake decrease [42], while resting and exercise blood pressure increase. Reaction time tends to slow, while muscular strength and flexibility decrease. In most individuals, body fat increases, glucose tolerance decreases, and recovery time increases [42]. Chronic conditions that may decrease an individual’s ability to acclimate to extreme heat include heart disease, vascular disease, chronic kidney disease, dementia, and diabetes [62].

Cognitive declines associated with aging are in part related to neurologic changes, including reductions in the volume of gray matter, changes in white matter connectivity between the prefrontal cortex (associated with decision-making) and posterior areas associated with sensory input and motor control [5]. The rate of change depends not only on genetics and the neurological changes described above, but also on levels of physical activity, nutrition, medications utilized to treat other chronic health conditions, and, in some cases, various forms of mental illness such as stress, anxiety, and depression [38]. Symptoms of cognitive decline include slowed reaction time, changes in attention, and changes in memory. In many cases, these symptoms remain unrecognized or undiagnosed, particularly in adults who are socially isolated. Increased social isolation among older adults has been associated with elevated risks of dementia and disability [48], making them more vulnerable to environmental threats, including heat-related illness.

### 4.2. Heat-Related Illness Pathophysiology in Older Adults

#### 4.2.1. Physiology of Heat Stress, Heat Strain, and Heat-Related Illnesses

Heat-related illness (HRI) is a term used to describe a spectrum of syndromes that result from disruptions in thermoregulation among some individuals exposed to extreme heat [4]. Heat stroke (classical and exertional) is the most serious condition and is considered a medical emergency. Other conditions that fall under the HRI umbrella include heat edema (swelling of the extremities), heat-induced muscle cramps, and heat exhaustion [4].

In most circumstances, the human body does an exceptional job of maintaining a homeostatic internal body temperature of about 37 °C (98.7 °F). Thermoreceptors in the skin’s dermis layer send signals to the brain, which utilizes autonomic and behavioral pathways to create a plan for action [54]. When the body senses rising temperatures, blood moves toward the skin through vasodilation, raising skin temperature and the potential for heat loss by convection (movement of air across the body surface) and radiation (heat radiating out from the body into the atmosphere). In addition, sweating allows heat to evaporate off of the body surface [54].

As people age, their bodies are less effective at thermoregulation [14]. Sensitivity to heat decreases, which slows down autonomic reactions to cool the body [65], and there is a delayed threshold for sweating, so the body is less effective at evaporative cooling [49]. In addition to reduced sensitivity to heat, the sweat glands themselves undergo changes (sweat gland atrophy) [16]. Total body water decreases with aging, making older adults more susceptible to dehydration during heatwaves [78]. Aging adults lose subcutaneous fat, reducing insulation against heat and cold. Reduced cardiac output reduces the ability to redirect blood to the skin’s surface, and the capillaries in the skin itself undergo aging-related changes as well. These physiologic changes that occur during healthy aging form the inner hub of our socio-ecological framework.

Physical function declines include reduced proprioception, which is the body’s ability to sense its position in space, and balance, along with muscle loss. Loss of balance combined with muscle weakness increases the risk of falling, which can have severe consequences in extreme heat. Contact burns from pavement are increasing in frequency, particularly in hot urban areas of the US desert southwest [45]. Mobility to prevent falls or limit prolonged pavement contact may be problematic, making aging adults more vulnerable to contact burns, with increasing severity as contact time with hot pavement increases [34]. The longer a person lies on a hot surface, the greater the potential for burns to reach beyond the epidermis [34], disrupting the protective barrier and increasing morbidity and mortality from infection and multiorgan dysfunction [68].

#### 4.2.2. Effects of Medications on Heat Tolerance

Many older adults take prescription medications to manage chronic diseases, including cardiovascular disease, diabetes, kidney disease, affective disorders (stress, anxiety, and depression), thyroid replacement, etc. [8]. In addition, over-the-counter medications, including NSAIDs, and alcohol use may also reduce the body’s ability to tolerate heat [18,44]. When individuals become dehydrated, the ability to clear medications out of the body is reduced [18]. Finally, certain sedatives, including opiates, benzodiazepines, antipsychotics, antidepressants, and anticonvulsants, may reduce thirst sensation and affect balance, which increases the risk of falls [18]. Beta blockers taken for cardiac conditions reduce dilation of superficial blood vessels, reducing the body’s ability to dissipate heat [64]. Diuretics, beta blockers, calcium channel blockers, antacids, laxatives, and lithium can lead to electrolyte imbalance, particularly in excessive heat. Psychiatric medications, including serotonin reuptake inhibitors, antipsychotics, and tricyclic antidepressants, impair sweating and hence cooling [18]. Finally, antipsychotics and anticholinergics interfere with central thermoregulation [18].

#### 4.2.3. Classical and Exertional Heatstroke

Two types of heatstroke are now recognized: classical heatstroke characterized by hot, dry skin with a rectal temperature of over 40 °C (104 °F), leading to confusion, loss of consciousness, and convulsions [54], and exertional heatstroke brought on by intense physical activity in extreme heat, characterized by sweating, rhabdomyolysis, rapid breathing and heartrate, and elevated core temperatures over 40 °C (104 °F). Classical heatstroke is the more deadly condition [17], due in part to its insidious onset [23]. Because heatstroke is a medical emergency, it is important that physicians speak with patients at heightened risk about precursor symptoms, which may include fatigue, nausea, headache, confusion, or giddiness [54]. Primary care providers or pharmacists can counsel patients on any prescription drugs taken for chronic conditions that can increase the risk for heatstroke. This includes loop diuretics, anticholinergics, anxiolytics, certain antidepressants and antipsychotics, beta blockers, and calcium channel blockers [3].

#### 4.2.4. Contact Burns

In extremely hot climates, falls on naturally heated surfaces, such as streets, sidewalks, or other paved areas, can lead to contact burns [45]. Surface temperatures ranging from 95–100 °F are hot enough to cause such burns. Burns are classified by the depth of penetration into the skin, with the depth of the burn dictating the type of medical treatment required [54].

### 4.3. Social and Behavioral Determinants of Heat Vulnerability

#### 4.3.1. Impact of Race, Ethnicity, Economic Status, and Sexuality

Among older adults, race, ethnicity, income, and sexuality may increase vulnerability to heat-related illness. Persons of color, those living in poverty, disabled individuals, and those lacking health insurance have been shown to have increased mean exposure to extreme heat, in a study of the contiguous US [7]. Some communities of color reside in built environments with more heat reflective surfaces [39]. In addition, ethnic minorities are often forced to work in higher-risk outdoor occupations, live in substandard housing, and have lower levels of education [43]. Immigrants and refugees, who may not be eligible for public benefits, including healthcare, are also more vulnerable [39]. Older adult immigrants or refugees who may have limited or no knowledge of English must overcome language barriers in addition to their health and housing needs. The population of incarcerated older adults in the US grew 282% between 1995 and 2010. These individuals are completely dependent on state, local, and federal government resources to ensure their welfare, including emergency preparedness [39]. While studies on LGBTQ individuals have not focused on susceptibility to heat illness, studies do point to higher levels of poverty [39].

#### 4.3.2. Behavioral Factors Influencing Heat Illness Vulnerability

Public education can only be effective if the people to whom this education is directed engage and take proactive measures. A community-level mixed-methods study of older adult residents in Waterloo, Canada [25], included 15 qualitative interviews, followed by quantitative surveys distributed to 225 predominantly female residents ages 52–97 via email, in person at community events, and through community partner agencies. Most participants lived independently in the community, and almost half (48.8%) lived alone. Most participants were aware of climate change, particularly heat waves, and the potential health-related risks. Higher perception of risk was associated with lower income. In addition, individuals living in apartments rather than houses believed they were at higher risk from extreme heat. Finally, the fewer the resources residents believed were available to them, the higher their perceived risk of heat vulnerability.

Researchers noted gaps in participants’ knowledge of heat illness symptoms. In addition, some were hesitant to utilize existing resources for fear of social stigma and being perceived as vulnerable. Other risk factors included social isolation, lack of social support, and lack of access to cooling. There were also communication gaps, particularly that some respondents were unfamiliar with the metric system and the differences between Celsius and Fahrenheit measurements. Finally, researchers recommended avoiding terminology such as “elderly” and “vulnerable,” which these individuals found stigmatizing [25].

### 4.4. Integrating Findings Within a Socio-Ecological Framework

#### Socio-Ecological Framework

**Figure 1:**
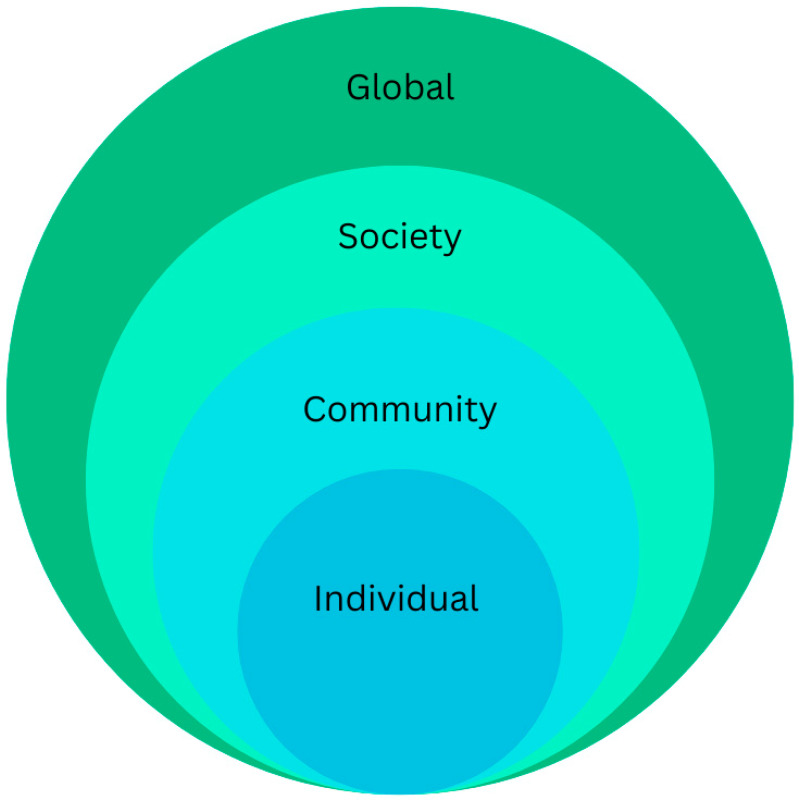
Socio-ecological framework for climate change and heat-related illness.

**Figure 2:**
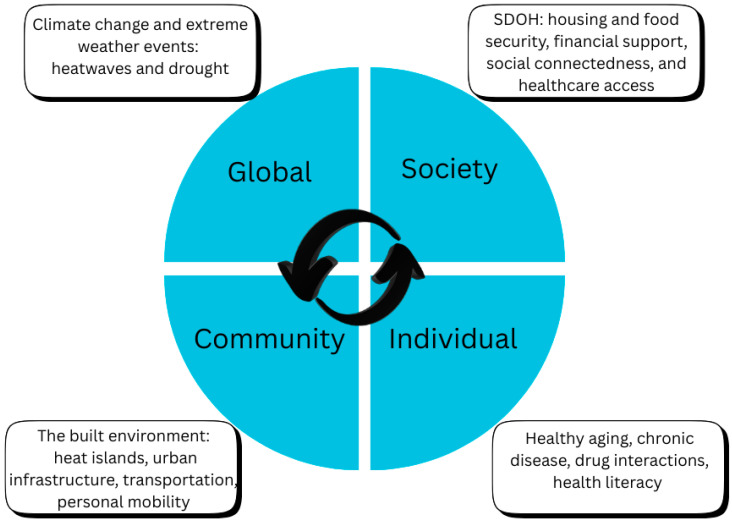
Drivers of heat-related illness in older adults.

The socio-ecological framework contextualizes heat-related illness, its drivers, and strategies to address the problem, at the individual, community, society and global levels. Within this framework, global warming (rising temperatures) represents the outer ring (global level), while processes of healthy aging and chronic disease in older adults form the inner hub (individual level). Middle layers include the built environment (community) and social determinants of health (society). Figure 1 depicts the basic framework, and Figure 2 depicts subcategories within each level. While global warming, aging processes, and social determinants are conceptualized within separate levels, it is important to keep in mind that they are interactive, a factor underlying many challenges in addressing heat-related illness at the population level. Although healthcare providers can address physiological and pathological processes underlying HRI, environmental factors described in the sections below are equally impactful.

### 4.5. Environmental and Structural Drivers of Heat Risk

#### 4.5.1. Global Warming

Average temperatures in the US have increased by 1.3° to 1.9 °F since record-keeping began in 1895, with the greatest degree of change occurring since 1970 [21]. Global temperatures have increased 1.45 °C above the pre-industrial average [8], with land areas warming faster than oceans [1]. While limiting greenhouse gas emissions will slow the pace of global warming, temperatures are expected to increase by 2.1 °C to 3.5 °C by the end of the 21st century [29].

#### 4.5.2. Extreme Weather Events

Heatwaves describe greater than or equal to 2 or 4 consecutive days when average daily temperatures exceed the 97th, 98th, or 99th percentile for a geographical area [37]. High ambient temperatures contribute to heat stress and heat illness, along with air velocity, humidity, and radiant temperature [54]. The frequency and severity of droughts are also expected to increase [73], impacting the supply and availability of drinking water, air quality and wildfire risk [21]. A study using Berkeley Earth data found that the number of heatwave days, particularly in low-latitude areas, has increased by as much as 3–5 days per decade, and that heatwave length has also increased, in some cases by over one day per decade [55]. Because many of the poorest nations in the world, such as those in sub-Saharan Africa, are in low latitudes, these populations are significantly more affected than wealthier nations in mid-latitude climates [34].

#### 4.5.3. The Built Environment: Urban Heat Islands

The urban heat island effect refers to elevated temperatures in cities compared to surrounding rural areas, attributable to urban construction and human activity [74]. In large urban areas such as New York (US) and Beijing (China), urban heat islands contribute to increases in ambient temperatures, which compound the effects of global warming [74]. Housing structures may have inadequate insulation to withstand temperature extremes. For example, mobile homes have come under fire for what is referred to as ‘thermal insecurity,’ [79], with a debate as to whether the inability of these types of homes to adequately maintain habitable temperatures during extreme heat is due to the materials and methods used in their construction, or is socially motivated [22]. Where a person lives within a multi-story building structure can also impact the interior environment. During the Chicago heat wave of 1995, elderly residents living on upper floors of multi-story buildings were among the most vulnerable, not only because heat rises, making higher residences hotter, but also because of increasing social isolation and limited mobility. Among the 465 deaths certified by the Cook County Medical Examiner’s office for being heat-related, 56% were age 75 or older [19].

#### 4.5.4. Social Drivers of Inequality

Social drivers, such as housing and financial insecurity, contribute to the vulnerability of older adults, many of whom are on fixed incomes, to heat-related illness. These individuals may not be able to afford the utility costs to maintain adequate cooling within their homes [62]. Lack of access to transportation, cooling centers that are inaccessible or unsafe for older adults, as well as lack of access to healthcare services, poor nutrition, and social isolation, all contribute to increased susceptibility to heat-related illness. A study analyzing data from 35,000 adults over age 50 revealed that frequent exposure to extreme heat resulted in disability progression in the ability to perform instrumental activities of daily living (IADLs) [41]. This same study found that functional declines were greatest among those living alone and not working.

### 4.6. Population Health Strategies and Risk Reduction

#### 4.6.1. Risk Reduction Strategies

**Figure 3:**
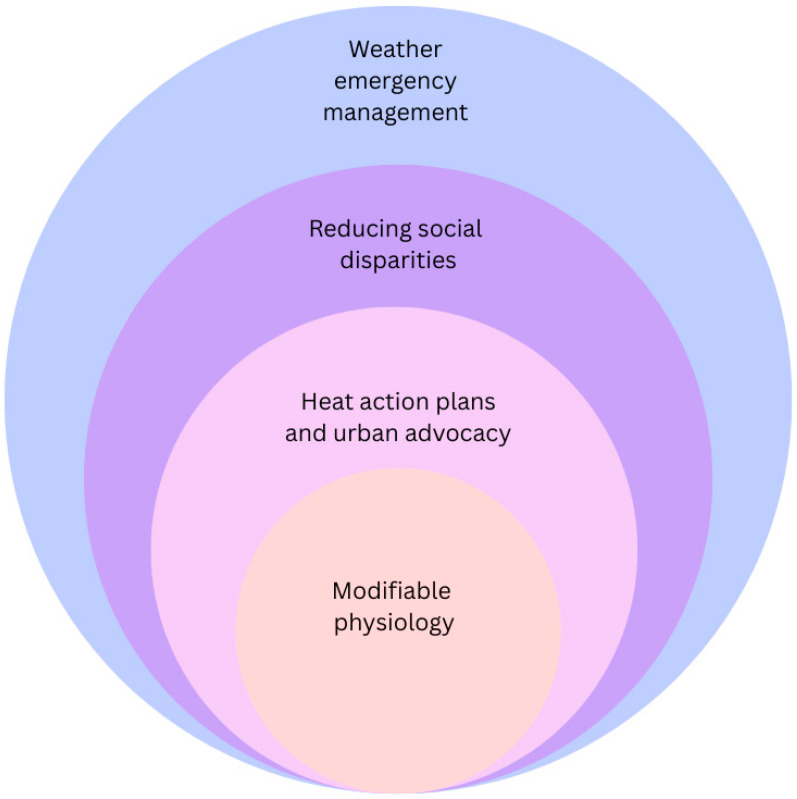
Risk reduction for heat-related illness within a socio-ecological framework.

Just as the drivers of heat-related illness among older adults exist along a continuum from the individual to the global levels, so do risk reduction strategies. Figure 3 depicts strategies to mitigate the impacts of climate change within our socio-ecological framework.

At the global level, considerations include measures to slow the rate of climate change and the efficacy of warning systems in motivating behavior change. The Paris Agreement, ratified by a majority of United Nations members in 2016, set the ambitious goal of minimizing the rise in global temperatures to no more than 1.5 °C above pre-industrial levels. However, the global economic impacts of COVID-19 and a changing political climate in the USA have made fulfillment of this agreement unlikely.

Public education is instrumental in driving policy change. A complex inter-generational relationship between the aging and younger generations affects the sense of ownership and efficacy in addressing both climate anxiety and risk mitigation. According to one report, natural disasters over the past decade have been responsible for 60,000 deaths annually worldwide [56]. That same report estimates economic damage to the USA due to greenhouse gas emissions between 1990 and 2014 at 2 trillion dollars, with China following close behind [56]. It is important that affected populations are aware of these statistics in order to promote policy change.

#### 4.6.2. Heat Warning Systems

Evidence is mixed regarding the efficacy of public warming systems for extreme weather events. A study of the impact of heat emergency alerts issued by the National Weather Service in 20 US cities found that these alerts were not associated with lower mortality rates [70]. A study of heat health warning systems in Shanghai based on data collected from the Chinese Center for Disease Control and Prevention found that 50% of heat-related illnesses and 58.2% of heat-related deaths in the city occurred on days when there were no heat warnings, questioning the usefulness of warnings based on a single metric: a temperature threshold of 35 °C [72]. Research on emergency department admissions during heat alerts found a higher rate of hospital admissions for fluid and electrolyte disorders and heatstroke, suggesting that heat alerts may lead more individuals to access care [71]. In addition to emergency alerts, heat response plans could include education on behavior change to prevent serious heat-related illness, as well as making resources (publicly distributed water, mobility support, cooling centers, etc.) available to vulnerable populations who lack access to air conditioning.

#### 4.6.3. Heat Action Plans

Increases in global temperatures have motivated municipalities globally to create heat action plans [15,35,63]. The motivation is to be proactive rather than reactive, utilizing risk assessments and long-term planning to prevent morbidity and mortality during heat waves [15]. This may include heat health watch warning systems [35], education for community health workers, and surveillance of vulnerable populations, in addition to structural changes such as cooling centers, public drinking fountains, etc. In some cases, these plans have successfully reduced morbidity and mortality during heat emergencies. For example, the PHASE program in Europe [35] not only provided community-level education and creation of cooling centers but also engaged physicians in surveillance of vulnerable subgroups. A program in France (2006) also focused on vulnerable populations and retirement homes, significantly reducing mortality during subsequent heat events [35]. A program in Canada in the early 2000s included telephonic check-ins with patients in hospitals and home care facility residents, reducing deaths by 2.52 persons per day compared to the years immediately preceding the intervention [35].

Oversight of heat action plans varies by location, with some plans developed by cities [63] and others by national governments [15]. For example, in the UK, the National Adaptation Programme aims to prepare for extreme weather events [15], while in India, such planning is handled within cities [63]. Unfortunately, while some plans have been successful, others lack forward-looking decision-making [15,63], conceptualizing extreme weather events as exceptions rather than (increasingly) the norm.

#### 4.6.4. Modifications to the Built Environment

Population health and urban planning strategies may reduce the vulnerability of elderly residents living in heat islands. This includes public green spaces, compliance with ADA requirements for mobility (curb cut-outs, ramps with proper grading for wheelchair access, proper door width for wheelchair access, etc.), access to social workers and caregivers, educating the public about renter’s rights for air conditioning maintenance/repair, and warnings about increased risks of overheating due to poor insulation in prefabricated homes. These measures enable behavior change to protect vulnerable individuals from the adverse effects of extreme heat. While most studies of urban green spaces focus on their cooling effects, they also contribute to social capital by contributing to social connectedness among socially isolated individuals, facilitating social trust and reciprocity, social participation, and positive identity with the place of residence [20]. Social capital from the incorporation of green spaces may build advocacy for other modifications within the built environment, such as safe and accessible cooling centers, transportation systems that account for the needs of individuals with physical disabilities, and walking paths within cities maintained to be free of tripping and falling hazards.

#### 4.6.5. Universal Design and Personal Mobility

Personal mobility is challenging for many older adults due to changes in vision, hearing, balance, and situational awareness, as well as the loss of muscle mass (sarcopenia). Universal design refers to structuring the environment so that it can be accessed, understood, and used by all people regardless of their age, size, ability, or disability [31]. In the USA, many of the principles of universal design, including equitable use, flexibility in use, simple and intuitive use, perceptual information, tolerance of error, low physical effort, and size and space for approach and use, are folded into the Americans with Disabilities Act of 1990 [13]. Given that many older adults have challenges with driving, it is particularly important to facilitate accessibility to public transportation and ambulation. This requires adequate lighting on streets and pedestrian pathways, continuous and stable pavement, sufficient maneuvering space on sidewalks, easily interpretable signage, removal of steps and small obstacles, inclusive crossings, noise pollution reduction, and protected level changes [57].

#### 4.6.6. Heat Safety Education

Public education is a critical population health strategy for reducing heat-related illness among older adults. Educational efforts should focus on increasing awareness of heat illness warning signs, when to seek medical care, and practical strategies to reduce exposure, such as modifying physical activity to cooler times of day. Public health agencies, healthcare providers, and community-based organizations serving older adults can disseminate this information through targeted outreach, including clinician counseling, senior centers, aging services networks, and localized heat alerts. Education should also address hydration strategies, as older adults are particularly vulnerable to dehydration and electrolyte imbalances during extreme heat due to age-related declines in total body water and impaired thirst response. One study reported dehydration prevalence among community-dwelling older adults ranging from 1% to 60%, depending on measurement methods [78], underscoring the importance of clear, actionable messaging on adequate fluid intake. For older adults with mobility limitations, education should include risk-mitigation strategies such as using assistive devices with seating, carrying a mobile phone, and arranging accompaniment from family or friends during extreme heat events.

#### 4.6.7. Interior Environments

An individual’s ability to acclimate depends both on physiology and behavioral changes enacted to adjust to a new environment. Control of the interior environment is critical, since most individuals spend the majority of their time, about 90%, indoors [28]. Recommended interior temperatures for older adults should not exceed 25 °C (77 °F). In addition to thermoregulation, this is the recommended temperature for storing medications [54].

Obtaining timely service for poorly functioning or nonfunctional air conditioning units can be a challenge for renters. In the US, renters’ rights are under state control [27], while in Europe they are controlled at the national level [59]. Older adults need to understand their rights as renters and have access to affordable legal services should a dispute with the landlord arise. Poorly functioning HVAC systems in prefabricated or mobile homes are another challenge. Mobile homes tend to be energy-inefficient due to poor insulation, which is particularly evident during heat waves and cold snaps. In the US, certain states, including Oregon, New York, and Maine, have implemented replacement initiatives that offer low-cost loans to eligible residents for replacing their outdated mobile homes with newer, energy-efficient units.

#### 4.6.8. Addressing Health-Related Social Needs

Social factors that impact an individual’s health include income, education, employment, social support, and culture [60]. In the USA and Europe, many older adults live on fixed incomes from pensions, retirement, and Social Security [51]. Assets from government-funded programs in Asia vary widely, with some older adults dependent primarily on retirement accounts and support from their children [26]. As the cost of living rises due to inflation, these individuals are challenged to make their monthly payments cover living expenses, along with any medical expenses not reimbursed by insurance. These expenses put individuals on fixed incomes at risk of food insecurity [41]. Between 2000 and 2015, the number of adults in the US who were food-insecure more than doubled to 5.4 million [41]. This, in turn, may exacerbate chronic diseases such as diabetes, which in turn makes these individuals more susceptible to heatstroke and other heat-related illnesses [54].

Physicians should be aware of the impact prescription drug costs have on their patients’ quality of life, food and housing security. While generic drugs represent 89% of drug prescriptions filled in the US, they account for only 26% of drug costs [80]. Whenever possible, generic substitutes should be prescribed [75]. While issues of food security may be outside the scope of practice, Medicare Part B in the US covers nutritional therapy services for individuals with diabetes, chronic kidney disease or kidney transplants [81].

Social isolation is defined as an objective lack of social contact with friends, family, and the community [11]. A systematic review and meta-analysis of 41 studies on the global prevalence of social isolation found that 26% of community-dwelling older adults are socially isolated [11]. Within the US, about a quarter of community-dwelling older adults are socially isolated [50]. Social isolation has been associated with statistically or near statistically significant worse prognosis in a study of 225 older adult patients with classic heat stroke [69]. A time-series multi-community series study of heatwave-related mortality risk in older adults between 2008 and 2017 found a lower heat-related mortality risk among older adults (age 65+) living in areas with higher social networks, meaning higher percentages of social gathering, mutual trust, mutual aid, and living in detached housing (in the community) [82].

Given its prevalence among older adults and its impact on morbidity and mortality, the National Academy of Sciences, Engineering and Medicine [50] recommends screening for social isolation within primary care. In medical homes with integrated behavioral health, screening can be conducted by the behavioral health consultant (BHC), who can work with patients to develop strategies for increasing socialization. Validated screening tools for social isolation include the Berkman Syme Social Network Index, Revised UCLA Loneliness Scale, Steptoe Social Isolation Index, and Duke Social Support Index [50].

For those without access to air conditioning, the Centers for Disease Control recommends skin wetting and using electric fans as alternative strategies. When temperatures permit, the CDC recommends opening doors and windows to allow fresh air to ventilate through the interior [83]. However, research shows that circulating hot, dry air, including the use of electric fans, can worsen heat stress in temperatures above 32.2 °C (90.0 °F). The use of fans for cooling can be particularly dangerous in dry environments [6], whereas skin wetting without electric fans may be beneficial in hot, dry environments.

### 4.7. Implications for Low- and Middle-Income Countries

#### Community Planning in Low-and-Middle-Income Countries

To date, research on this topic remains sparse, specifically regarding older adult populations. A systematic review [61] reported that one city (Ahmedabad, India) had developed an early warning system based on collected evidence. Within rural areas that may lack brick-and-mortar healthcare facilities and licensed physicians, community health workers could play an important role in patient education and emergency preparedness. While information exists regarding such efforts, the focus tends to be on the general population with no specific focus on older adults. For example, a cluster-randomized controlled trial in Karachi, Pakistan, utilized an educational intervention delivered by CHWs to help community members reduce their risks of adverse reactions to heat and recognize the early signs of heat illness [58]. While this was a well-researched and documented study, the focus on randomization was on different ethnic groups rather than the ages of the participants. A separate study examining the role of CHWs in delivering health services and patient education around heat-related illness included numerous LMICs in Africa, Asia, and the Western Pacific [24]. However, the only age group mentioned specifically was children. In addition, this review covered a range of extreme weather events, in addition to heat waves, such as flooding and landslides.

A systematic review [42] of community-based interventions to improve heat preparedness included ten studies, of which two were conducted in LMICs. Interventions included educational pamphlets, videos, telephonic outreach, face-to-face meetings, and, in one case, individual care plans (Italy). However, once again, age groups were not identified in the study.

## 5. Conclusions

Rapid increases in the rate of global warming, together with the aging of the global population, could result in a “perfect storm,” in which older adults have neither the knowledge nor the resources to effectively acclimate. While neither climate change nor shifts in population demographics are readily reversible, numerous strategies exist to help the growing number of adults over age 65 adapt. Heat action plans in North America and Europe are an important step in improving population-level education about global warming, heat-related illness, and resources within the built environment, healthcare systems, and individual-level behavioral changes to reduce risks. As the population of older adults increases in developing nations, it will be particularly important to implement similar planning in these areas. The use of community health workers to help with education and the navigation of existing resources is especially important in developing nations. Future research should investigate the role of physical activity and nutritional strategies to build resilience, strategies for patient and public education, and opportunities for policy change.

## Figures and Tables

**Table 1:** Sources extracted, including the authors’ countries of origin.

Authors and Publication Date	Country of Origin	Primary Focus
ADA.gov [13]	USA	Standards/Accessible design
Anu et al., 2023 [14]	South Africa	Effects of climate change on older adults
Arnell, 2022 [15]	UK	Implications of climate change for emergency planning
Balmain et al., 2018 [16]	Australia	Aging and thermoregulatory control
Bouchama et al., 2022 [17]	Saudi Arabia	Classical and exertional heatstroke
Cabeza et al., 2018 [5]	USA	Neuroscience of healthy aging
CDC, US, 2005 [18]	USA	Interaction of medications with heat
CDC, US, 1995 [19]	USA	Chicago heatwave of 1995
Chaseling et al., 2024 [6]	USA	Reduction of cardiac strain on older adults in extreme heat
Chow et al., 2012 [12]	USA	Heat island effect, policy implications
Clark et al., 2024 [7]	USA	Demographic groups at risk from extreme heat
Cornu et al., 2024 [20]	Belgium	Urban green spaces and mortality among older adults
Crimmins et al., 2016 [21]	USA	Impacts of climate change on human health in the US
Curran-Groome et al., 2025 [22]	USA	Mobile homes, climate extremes and policy
Dalip et al., 2015 [23]	Australia	ED visits by older adults during an Australian heat wave
Domingo et al., 2024 [24]	Canada	Role of CHWs in heat-related illness in LMCs
Eady et al., 2016 [25]	Canada	Mixed methods study of perceptions about heat illness among older adult residents in Waterloo, Canada
East West Center, 2020 [26]	USA	Economic challenges from an aging population in Asia
Environmental Law Institute, 2017 [27]	USA	Indoor air quality guide for renters
Environmental Protection Agency, 2022 [28]	USA	Safety of the indoor environment
European Environment Agency, 2025 [29]	Denmark	Global and European temperature trends
Fuster, 2017 [30]	USA	Changes in global healthcare due to the aging of populations
Gauer & Meyers, 2019 [4]	USA	Explanation of heat-related illness
GSA, Section 508, n.d. [31]	USA	Universal design
Harrington et al., 1995 [32]	USA	Pavement burns
Harrington et al., 2016 [33]	New Zealand	Temperature extremes in low-and-middle-income nations
Harrington & Otto, 2023 [34]	New Zealand	Underestimated climate risks from population aging
Hasan et al., 2021 [35]	Pakistan	Community-based interventions for prevention of heat-related illness
Hong et al., 2023 [36]	China	Responses of global health systems to challenges associated with population aging
Hopp et al., 2018 [37]	USA	Medical diagnoses for patients admitted to hospitals for heat-related illness
Hunter et al., 2016 [38]	USA	Behavioral health considerations for older adults
Ica.Ca.gov, n.d. [39]	USA	Overview of vulnerable populations for heat-related illness
Jih et al., 2018 [40]	USA	The relationship between chronic disease and food insecurity among older adults
Ji et al., 2025 [41]	USA	Functional disability among socially isolated older adults
Johar, H. et al., 2025 [42]	Malaysia	Community-based heat adaptation interventions
Jung et al., 2021 [43]	USA	Use of heat illness data to create vulnerability maps in the US
Kenny et al., 2010 [44]	Canada	Heat stress in older adults with chronic medical conditions
Kowal-Vern et al., 2019 [45]	USA	Contact burns
Layton et al., 2020. [46]	USA	Heatwaves and hospitalizations in persons with chronic illness
Lewis, 2007 [3]	USA	Heatstroke in older adults
Liguori et al., 2022 [47]	USA	Physiological effects of aging in relation to extreme heat
Lyu et al., 2022 [48]	USA	Social isolation and health outcomes in older adults
Meade et al., 2022 [49]	Canada	Hemodynamic regulation during heat exposure: comparison between younger and older adults
National Academies of Sciences, Engineering and Medicine, 2020 [50]	USA	Health effects of social isolation and loneliness in older adults
National Council on Aging [51]	USA	Effects of fixed income on the quality of life for older adults
Nocentini, 2024 [52]	Italy	Metropolitan adaptation plans for climate change
Núñez-Rodriguez et al., 2025 [53]	Spain	Heat tolerance in older adults
Parsons, K., 2019 [54]	USA	Physiology of human heat stress
Perkins-Kirkpatrick & Lewis, 2020 [55]	Australia	Trends in regional heatwaves
Raihan, 2023 [56]	Malaysia	Impact of global climate change and adaptation strategies from socio-economic perspectives
Ramirez-Saiz et al., 2025 [57]	Spain	Improving urban mobility for older adults
Razzak et al., 2022 [58]	Pakistan	Role of CHWs in education and heat emergency preparedness
RentersRightsLondon.org [59]	London, UK	Renter’s rights in Europe
Romanello et al., 2024 [8]	London, UK	Health and climate change
Russo et al., 2019 [60]	USA	Health care policy in the US
Sapari et al., 2023 [61]	Malaysia	Impact of heat waves on health care in LMICs
Sarofim et al., 2016 [62]	USA	Temperature-related death and illness
Singh et al., 2024 [63]	India	Urban heat action plans in India
Sorenson & Hess, 2022 [64]	USA	Prevention and treatment of HRI
Takeda et al., 2016 [65]	Japan	Lowered thermal sensation in normothermic older adults
Teo et al., 2023 [11]	Singapore	Global prevalence of social isolation among older adults
USDHHS, n.d. [66]	USA	Optimal health standards for US adults
USGS, n.d. [67]	USA	US Geological Service definitions of global warming and climate change
Vardy et al., 1989 [68]	USA	Contact burns
Wang et al., 2023 [69]	China	Survivability of heatstroke
Weinberger et al., 2018 [70]	USA	Role of national weather service heat alerts in population health
Weinberger et al., 2021 [71]	USA	Heat warnings, mortality and hospital admissions in the US
World Health Organization, 2025 [2]	Switzerland	Aging and health
Wu et al., 2020 [72]	China	Performance of heat health warnings in Shanghai
Wu et al., 2014 [9]	USA	ED visits for heat stroke in the US
Xu et al., 2019 [73]	China	Global drought trends
Yang et al., 2016 [74]	China	Urban heat island effect
Zhou et al., 2022 [75]	USA	Out-of-pocket costs for prescription medications
Zhu et al., 2025 [10]	China	Chronic disease prevalence in older adults
Zubaishvili & Zubaishvili, 2021 [76]	Georgia	Population aging as a global challenge

**Table 2:** Physiology, chronic disease, heat-related illness and related risks.

Authors, Date	Type of Study or Source	Focus
Balmain et al., 2018 [16]	Review	Thermoregulatory control and heat stress during exercise among older adults.
Bouchama et al., 2022 [17]	Review	Classical and exertional heatstroke
Cabeza et al., 2018 [5]	Review	Cognitive neuroscience of healthy aging: maintenance, reserve and compensation
CDC, US, 2005 [18]	US government website	Impact of medications on heat tolerance
Chaseling et al., 2024 [6]	Correspondence	Strategies to reduce cardiac strain in older adults in extreme heat.
Crimmins et al., 2016 [21]	US Global Change Research Program	Impacts of climate change on vulnerable populations
Dalip et al., 2015 [23]	Retrospective case–control study	5-year study of ED admissions during heat waves in an Australian metropolitan hospital
Fuster, 2017 [30]	Editorial	Cardiologist’s perspective on emerging healthcare needs of older adults
Gauer & Meyers, 2019 [4]	Medical education	Heat-related illnesses
Harrington et al., 1995 [32]	Retrospective case series	Study of 23 patients who developed contact burns from pavement in the Southwestern US.
Ica.Ca.gov, n.d. [39]	Executive summary	State of California executive study on the impacts of SDOH on heat illness vulnerability
Hopp et al., 2018 [37]	Review	Data from US Medicare enrollees: heat wave-related hospital admissions
Hunter et al., 2016 [38]	Book	Graduate-level textbook on behavioral health in primary care.
Jih et al., 2018 [40]	Secondary analysis of two national studies on older adults	Relationship between food insecurity and chronic disease in older adults.
Kenny et al., 2010 [44]	Review	Heat stress in older adults with chronic disease
Kowal-Vern et al., 2019 [45]	Retrospective data study	Contact burns from pavement among adults living in the US Southwest
Layton et al., 2020 [46]	Original research	US Medicare data on the impact of medications on the risk of HRI in older adults.
Lewis [3]	Review with case studies	Heatstroke in older adults
Liguori et al., 2022 [47]	American College of Sports Medicine book	Aging and physical activity
Meade et al., 2022 [49]	Clinical trial	Evaluation of body temperature and hemodynamic regulation following 9 h of heat exposure
National Academies of Sciences, Engineering and Medicine, 2020 [50]	NASEM report	Social isolation and loneliness among older adults
Núñez-Rodriguez et al., 2025 [53]	Systematic Review	Heat tolerance in older adults
Parsons, K., 2019 [54]	Book	
Romanello et al., 2024 [8]	Review	Global perspective on the human costs of climate change.
Sarofim et al., 2016 [62]	US Global Health Research Program	Effects of global warming on rates of morbidity and mortality
Sorenson & Hess, 2022 [64]	Perspective	Clinical risk reduction strategies for preventing heat-related illness
Takeda et al., 2016 [65]	Clinical trial	Comparison of normothermic and hyperthermic older adults to younger adults
Wang et al., 2023 [69]	Multi-center study	Prognostic model for survival of classical heatstroke
World Health Organization, 2025 [2]	Website	Global perspective on climate change and human health
Wu et al., 2014 [9]	Original research	Investigation of US hospital data of ED visits for heat stroke, 2009–2010
Zhou et al., 2022 [75]	Review	Out-of-pocket costs for prescription medications for chronic medical conditions
Zhu et al., 2025 [10]	Systematic review and meta-analysis	Almost half of older adults live with multiple chronic conditions

**Table 3:** Environmental factors influencing heat vulnerability among older adults.

Authors and Date	Type of Study or Source	Narrative Focus
ADA.gov, n.d. [13]	US government website	Accessibility guidelines for persons with physical disabilities
Anu et al., 2023 [14]	Review	Impact of housing insecurity, social support, temperature extremes and extreme weather events on older adults
Arnell, 2022 [15]	Review	Implications of climate change for emergency planning in the UK
CDC. US, 1995 [19]	Weekly morbidity and mortality report	Morbidity and mortality report on the 1995 Chicago heat wave.
Chow et al., 2012 [12]	Review	Urban heat island effect
Clark et al., 2024 [7]	Review	Effects of age, race, ethnicity and socioeconomic status on risk of HRI
Cornu et al., 2024 [20]	Review	Urban green spaces to reduce heat islands and improve socialization
Curran-Groome et al., 2025 [22]	Urban Institute Website article	Mobile homes are vulnerable to climate extremes
Domingo et al., 2024 [24]	Review	Role of CHWs in LMICs to manage population risks during extreme weather events
Eady et al., 2016 [25]	Mixed methods study	Attitudes of older adults living in Waterloo, Canada, about climate change
East West Center, 2020 [26]	Review	Economic challenges of increasing older adult populations in Asia
Environmental Law Institute, 2017 [27]	Article	Indoor air quality guide for tenants
Environmental Protection Agency, 2022 [28]	Infographic from the US EPA	Improving air quality in the indoor environment
European Environment Agency, 2025 [29]	Article	Increases in global and European temperatures between 1850 and 2020 due to industrialization
GSA, Section 508, n.d [31]	US Government agency website	Universal design
Harrington et al., 2016 [33]	Review	Poorest nations experience earlier emergence of daily temperature extremes
Harrington & Otto, 2023 [34]	Review	Climate risks for aging populations
Hasan et al., 2021 [35]	Review	Community-based interventions for management of HRI
Hong et al., 2023 [36]	China CDC perspectives article	Responses of global health toward population aging with a focus on international action plans
Ji et al., 2025 [41]	Review of data from 35,000 older adults	Impacts of functional disability and social isolation on risks for HRI
Johar et al., 2025 [42]	Systematic review	Community-based adaptation plans to mitigate the impacts of extreme weather events
Jung et al., 2021 [43]	Case-crossover study	Use of heat illness data in the state of Florida (US) to assess impacts of heat on human health
Nocentini, 2024 [52]	Systematic review	Analysis of metropolitan climate adaptation plans
Perkins-Kirkpatrick & Lewis, 2020 [55]	Review	Global and regional trends in heat waves from 1950 to 2000
Raihan, 2023 [56]	Research	Impacts of global climate change and adaptation strategies
Ramirez-Saiz et al., 2025 [57]	Review	Importance of universal design for social inclusion of older adults and people with disabilities.
Razzak et al., 2022 [58]	Randomized controlled trial	Community education to improve climate literacy
Russo et al., 2019 [60]	Textbook on health care delivery in the US	Population health approaches to health care delivery
Sapari et al., 2023 [61]	Systematic review	Impacts of heat waves on healthcare in LMICs
Singh et al., 2024 [63]	Review	Efficacy of heat action plans in cities in India
Teo et al., 2023 [11]	Systematic review and meta-analysis	Global study of social isolation among community-dwelling older adults
USGS, n.d. [67]	US government agency website	What is the difference between climate change and global warming?
Weinberger et al., 2018 [70]	Research using weather alert data from the US National Weather Service	Effectiveness of heat alerts in preventing mortality in 20 US cities
Weinberger et al., 2021 [71]	Review	Heat warnings and hospital admissions among older adults in the US, 2006–2016
Wu et al., 2020 [72]	Case study	Performance of heat health warning systems in Shanghai, China
Xu et al., 2019 [73]	Original research	Global drought trends due to climate change
Yang et al., 2016 [74]	Original research	Urban heat island effect
Zubaishvili & Zubaishvili, 2021 [76]	Review	Impact of global population aging from a social and policy perspective

## Data Availability

There were no data to report, only publicly available peer-reviewed data.
